# Secondhand Nicotine Absorption From E-Cigarette Vapor vs Tobacco Smoke in Children

**DOI:** 10.1001/jamanetworkopen.2024.21246

**Published:** 2024-07-11

**Authors:** Harry Tattan-Birch, Jamie Brown, Sarah E. Jackson, Martin J. Jarvis, Lion Shahab

**Affiliations:** 1 Department of Behavioural Science and Health, University College London, London, United Kingdom; 2 SPECTRUM Consortium, London, United Kingdom

## Abstract

**Importance:**

With the prevalence of e-cigarette use (vaping) increasing worldwide, there are concerns about children’s exposure to secondhand vapor.

**Objective:**

To compare nicotine absorption among children who are (1) exposed to secondhand tobacco smoke only or (2) exposed to secondhand vapor only with (3) those exposed to neither.

**Design, Setting, and Participants:**

The US Continuous National Health and Nutrition Examination Survey (NHANES) is a repeat cross-sectional survey. Participants are interviewed in their homes and, several days after, visit a mobile examination center to provide biological specimens. This study uses data from a nationally representative sample of US households from 2017 to 2020. Participants were children aged 3 to 11 years with serum cotinine levels incompatible with current firsthand nicotine use (ie, <15 μg/L). The final analysis was conducted on January 9, 2024.

**Exposures:**

Reported exposure to secondhand smoke or vapor indoors in the past 7 days (only secondhand smoke, only secondhand vapor, or neither). Covariates included age, sex, ethnicity, family income, body weight, and height.

**Main Outcomes and Measures:**

The primary outcome was serum cotinine concentration, an objective biomarker of nicotine absorption. Geometric mean cotinine levels and 95% CIs were calculated using log-normal tobit regression, accounting for the complex survey design and weights.

**Results:**

The mean (SD) age of the 1777 children surveyed was 7.4 (2.6) years, 882 (49.6%) were female, and 531 (29.9%) had family incomes below the poverty level. Nicotine absorption, as indexed by serum cotinine level, was highest among children only exposed to secondhand smoke (0.494 μg/L μg/L; 95% CI, 0.386-0.633 μg/L), followed by those exposed only to secondhand vapor (0.081 μg/L; 95% CI, 0.048-0.137 μg/L), equating to 83.6% (95% CI, 71.5%-90.5%; *P* < .001) lower nicotine absorption. Among children with no reported secondhand exposure, the geometric mean cotinine level was 0.016 μg/L (95% CI, 0.013-0.021 μg/L), or 96.7% (95% CI, 95.6%-97.6%; *P* < .001) lower than for those with exposure to secondhand smoke. Results were similar after covariate adjustment.

**Conclusions and Relevance:**

In this cross-sectional study of US children, nicotine absorption was much lower in children who were exposed to secondhand vapor vs secondhand smoke, but higher than in those exposed to neither. These findings suggest that switching from smoking to vaping indoors may substantially reduce, but not eliminate, children’s secondhand exposure to nicotine and other noxious substances.

## Key Points

**Question:**

How does children’s nicotine absorption, as indexed by serum cotinine level, differ among those (1) exposed to secondhand tobacco smoke only, (2) those exposed to secondhand e-cigarette vapor only, or (3) those exposed to neither?

**Findings:**

This cross-sectional study of 1777 US children aged 3 to 11 years found that, compared with children exposed to secondhand smoke only, nicotine absorption was 83.6% lower in those exposed to secondhand vapor only and 96.7% lower in those exposed to neither.

**Meaning:**

These findings suggest that children absorb much more nicotine from secondhand smoke than from secondhand vapor; switching from smoking to vaping indoors may substantially reduce children’s secondhand exposure to nicotine and other noxious substances, but both smoke and vapor increase children’s absorption vs no exposure.

## Teaser

This cross-sectional study uses serum cotinine level to compare nicotine absorption among children who are exposed to secondhand tobacco smoke only or exposed to secondhand vapor only vs those who are exposed to neither.

## Introduction

Children’s exposure to secondhand tobacco smoke has declined sharply in many countries over the past few decades, including the US and England, as parental cigarette smoking has decreased and those still smoking have increasingly opted not to smoke within the home.^1,2^ A growing proportion of people live in homes with smoke-free rules, which protects children from exposure to secondhand smoke.^2,3,4^ However, with the proliferation of nicotine e-cigarettes (ie, vaping) since the 2010s, there is another potential source of secondhand exposure to noxious substances in indoor air. It is especially important to investigate children’s secondhand vapor exposure because people who vape appear much more likely than those who smoke to do so indoors, even if they live with children.^5,6^

Cigarettes produce 2 sources of secondhand smoke: mainstream smoke (ie, smoke that a smoker inhales and then exhales into the environment) and side stream smoke (ie, smoke from the lighted end of a burning cigarette). Vaping does not generate aerosol between puffs, and laboratory studies^7,8^ show that vapers retain more than 99% of the nicotine they inhale. Therefore, children’s exposure to nicotine from secondhand vapor is likely to be substantially lower than that from secondhand smoke, and exposure to other harmful substances is likely to be lower still given that e-cigarette aerosol contains far fewer toxicants and carcinogens than tobacco smoke (and those that are remain are present in lower concentrations).^9,10,11,12^ This reduced exposure has been shown in artificial settings (eg, rooms with a smoking machine set up to either vape or smoke)^8,13^ and nonrepresentative samples,^14^ but to our knowledge no studies have examined exposure among children in natural settings using nationally representative samples of the population.

Although secondhand nicotine absorption itself is likely of limited risk, increased levels of nicotine biomarkers indicate that a child has also been exposed to other harmful constituents of tobacco smoke or e-cigarette aerosol. Therefore, using nationally representative data from the US Continuous National Health and Nutrition Examination Survey (NHANES), this study aims to compare nicotine absorption, as indexed by serum cotinine concentration, among children (aged 3-11 years) who do not use nicotine but are (1) exposed to secondhand smoke only or (2) exposed to secondhand vapor only, with (3) those exposed to neither.

## Methods

### Design

NHANES is a repeated cross-sectional survey that recruits participants of all ages in the US. It uses a multistage, complex probability sampling approach (described in detail in full NHANES methods report)^15^ to generate nationally representative information about the civilian population who live in households (noninstitutionalized). People who are Black or Hispanic are oversampled to allow for precise estimates of outcomes within these subgroups. We used data collected between 2017 and March 2020 (prior to disruption caused by the COVID-19 pandemic, which resulted in changes in the methods of NHANES).^15^

Participants completed the main interview in their homes. Several days after this, they were invited to a mobile examination center to provide a blood sample and answer several additional questions. A proxy respondent was asked to provide answers to the questionnaire for children aged 11 years or younger. The proxy respondent is the family member or legal guardian aged 18 years or older who knows most about the child (usually the mother or father). Response rates were 63% for 1- to 5-year-olds and 59% for 6- to 11-year-olds, of whom 92% and 89%, respectively, visited the mobile examination center. Survey weights were provided to account for nonresponse to interview and examination.

 Data collection for NHANES was approved by the National Center for Health Statistics Research Ethics Review Board. All participants’ parents or guardians gave written informed consent to take part in the study. This report follows the Strengthening the Reporting of Observational Studies in Epidemiology (STROBE) reporting guidelines for cross-sectional studies.

### Participants

Our analyses were restricted to children aged 3 to 11 years, because these individuals were unlikely to ever have used nicotine products (cotinine was unavailable for those aged 0-2 years), and 2 excluded children with cotinine levels more than 15 μg/L (to convert cotinine to nanomoles per liter, multiply by 5.675) in their blood serum, which suggested that they may have recently used a nicotine product.^16^ Thus, the included sample was children for whom their only source of nicotine would be from secondhand exposure from breathing in other people’s tobacco smoke or e-cigarette vapor (apart from the negligible amount absorbed from food).

### Outcomes and Measures

#### Reported Secondhand Exposure

To assess secondhand smoke exposure, the proxy respondent was asked, “Not counting decks, porches, or detached garages, how many people who live here smoke cigarettes, cigars, little cigars, pipes, water pipes, hookah, or any other tobacco product inside this home?” They were also asked whether the child was exposed to “someone else[s] smoke [from] cigarettes or other tobacco products indoors?” within the past 7 days across a number of possible locations (restaurant, workplace, bar, car, or in another person’s home). Children who live in a home where people smoke tobacco inside or who were present inside of the aforementioned locations when someone else was smoking were considered to be exposed to secondhand tobacco smoke.

To assess secondhand vapor exposure, similarly, the proxy respondent was asked, “During the last 7 days, was [the child] in an indoor place where someone was using an e-cigarette, e-hookah, vape-pen or other similar electronic product?” Children who were present inside any of these locations when someone else was vaping were considered to be exposed to secondhand e-cigarette vapor.

We compared children who were reported to have been exposed to (1) secondhand tobacco smoke only, (2) secondhand e-cigarette vapor only, or (3) neither. Children who were exposed to both secondhand smoke and secondhand vapor were excluded from the analysis because it was not possible to determine what proportion of their nicotine absorption was from the former vs the latter.

#### Covariates

The proxy respondent provided information on the child’s age, sex (male or female), and family income (below the poverty level, above the poverty level, or refused to answer, following Department of Health and Human Services’ poverty guidelines^17^). They also provided information on race and ethnicity, with responses categorized as Hispanic, non-Hispanic Black, non-Hispanic White, multiracial, or other (including American Indian, Alaska Native, Asian, Native Hawaiian, Pacific Islander, or any other race not otherwise specified). Body weight and height measurements were taken by a nurse during the child’s visit to the mobile examination center. These variables were included as covariates because they have been found to be associated with cotinine concentration in previous studies.^18^ Further details about these measures and methods are available elsewhere.^15^

#### Nicotine Absorption (Serum Cotinine)

The concentration of cotinine in body fluids has been used to quantify secondhand smoke exposure in population-based surveys since the 1980s.^19^ This is because, among people who do not use nicotine products themselves, cotinine is a precise and accurate marker of recent absorption of nicotine from secondhand smoke or vapor (ie, breathing in the tobacco smoke or e-cigarette vapor of others).

Blood samples were assayed at a single US Centers for Disease Control and Prevention laboratory to identify serum cotinine concentration using high-performance liquid chromatography/atmospheric-pression ionization tandem mass spectrometry, which has been validated in a multilaboratory international round-robin study.^20^ The lower limit of detection (LLD) of the cotinine assay was 0.01 μg/L. Tobit regression was used to account for children with undetectable cotinine levels.^1,3^

### Statistical Analysis

We used log-normal tobit regression models to estimate geometric mean levels of serum cotinine in children with (1) only secondhand smoke exposure, (2) only secondhand vapor exposure, and (3) no exposure. Models were repeated with covariate adjustment for age, sex, ethnicity, family income, body weight, and height.

Tobit regression was used to account for children with cotinine levels below the LLD of the assay. This assumes the residuals in cotinine levels are log-normally distributed, with censoring of values below the LLD of the assay (3% undetectable for children only exposed to secondhand smoke, 7% for only secondhand vapor, and 38% for neither). Because of the multistage complex survey design, the survey package in R statistical software version 4.3.1 (R Project for Statistical Computing) was used to account for the clustering within households and sampling units, nonresponse to interview, and oversampling of specific subgroups.^21^ This allowed for estimates that were broadly representative of the population of children who live in households in the US. A 2-sided α = .05 was used as the threshold for statistical significance. Data and analysis code are openly available on the Open Science Framework.^22^ Final analysis was conducted on January 9, 2024.

## Results

Two of the 1827 children (0.1%) surveyed were removed from the sample because they had serum cotinine concentrations that suggested they may have recently used nicotine products (>15 μg/L). Of the remaining 1825 children, 271 (14.8%) were reported to have been exposed to secondhand tobacco smoke only, 45 (2.5%) to secondhand e-cigarette vapor only, and 1476 (80.1%) to neither. After excluding 33 children (1.8%) who were exposed to both secondhand smoke and secondhand vapor and a further 12 children (0.7%) with missing body weight or height data, this left an analytic sample of 1777 children.

The mean (SD) age of participants was 7.4 (2.6) years, 882 (49.6%) were female, and 531 (29.9%) had reported family incomes below the poverty line. Similar proportions of the sample were Hispanic (477 children [26.8%]), non-Hispanic Black (463 children [26.1%]), and non-Hispanic White (519 children [29.2%]), whereas 318 children (17.9%) were multiracial or of other races and ethnicities. Table 1 shows demographic characteristics stratified by reported secondhand exposure.

**Table 1.** Demographic Characteristics of Children Aged 3 to 11 Years, by Secondhand Exposure to Tobacco Smoke and E-Cigarette Vapor

Characteristic	Participants, No. (%)			
	Exposed to smoke only (n = 270)	Exposed to vapor only (n = 43)	Exposed to neither smoke nor vapor (n = 1464)	Total (N = 1777)
Age, mean (SD), y	7.5 (2.4)	8.2 (2.6)	7.3 (2.6)	7.4 (2.6)
Sex				
Male	143 (53.0)	22 (51.2)	730 (49.9)	895 (50.4)
Female	127 (47.0)	21 (48.8)	734 (50.1)	882 (49.6)
Race and ethnicity				
Hispanic	37 (13.7)	3 (7.0)	437 (29.8)	477 (26.8)
Non-Hispanic Black	98 (36.3)	8 (18.6)	357 (24.4)	463 (26.1)
Non-Hispanic White	94 (34.8)	21 (48.8)	404 (27.6)	519 (29.2)
Multiracial or other^a^	41 (15.2)	11 (25.6)	266 (18.2)	318 (17.9)
Family income				
Above poverty level	123 (45.6)	34 (79.1)	891 (60.9)	1048 (59.0)
Below poverty level	125 (46.3)	7 (16.3)	399 (27.3)	531 (29.9)
Refused to specify	22 (8.1)	2 (4.7)	174 (11.9)	198 (11.1)
Body weight, mean (SD), kg	32.6 (14.1)	35.3 (16.6)	31.3 (14.6)	31.6 (14.6)
Height, mean (SD), cm	128.7 (16.2)	132.3 (17.4)	127.0 (17.8)	127.4 (17.6)

^a^ Other race and ethnicity includes American Indian, Alaska Native, Asian, Native Hawaiian, Pacific Islander, or any other race or ethnicity not otherwise specified. Data and analysis code are openly available online.^22^

The Figure shows that nicotine absorption, as indexed by geometric mean serum cotinine concentration, was highest among children who were exposed to secondhand smoke only (0.494 μg/L; 95% CI, 0.386-0.633 μg/L), followed by those exposed to secondhand vapor only (0.081 μg/L; 95% CI, 0.048-0.137 μg/L). This equates to 83.6% (95% CI, 71.5%-90.5%; *P* < .001) lower nicotine absorption for those exposed to secondhand vapor vs secondhand smoke. Children with no reported secondhand exposure had the lowest geometric mean cotinine levels (0.016 μg/L; 95% CI, 0.013-0.021 μg/L), or 96.7% (95% CI, 95.6%-97.6%; *P* < .001) lower than those with secondhand smoke exposure and 80.1% (95% CI, 64.9%-88.7%; *P* < .001) lower than those with secondhand vapor exposure. Thus, children exposed to secondhand vapor had 402% (95% CI, 185%-786%; *P* < .001) higher cotinine levels than those with no reported exposure. Similar results were found after covariate adjustment (Table 2). In children exposed to secondhand vapor only, slightly lower cotinine levels were found after excluding those who live with tobacco smokers from the analysis (eTable in Supplement 1).

**Figure.**
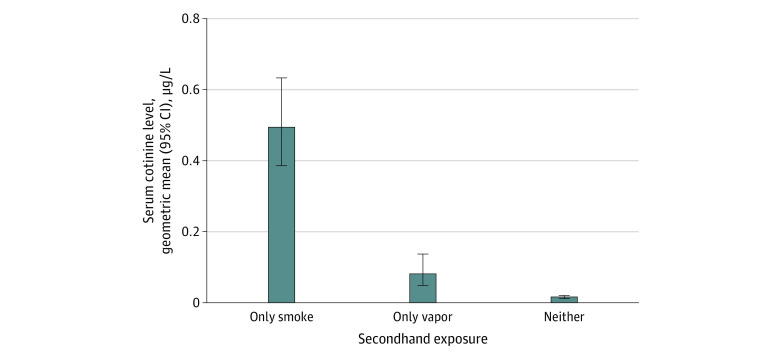
Nicotine Absorption by Reported Secondhand Exposure to Tobacco Smoke and E-Cigarette Vapor in Children Aged 3 to 11 Years Serum cotinine concentration is used as a biomarker of recent nicotine absorption. To convert cotinine to nanomoles per liter, multiply by 5.675. Estimates come from unadjusted log-normal tobit regression accounting for complex design of the National Health and Nutrition Examination Survey. Error bars represent 95% CIs. Data and analysis code are openly available online.^22^

**Table 2.** Nicotine Absorption by Reported Secondhand Exposure to Tobacco Smoke and E-Cigarette Vapor in Children Aged 3 to 11 Years

Secondhand exposure	Serum cotinine, μg/L^a^			
	Unadjusted GM (95% CI)	*P* value	Adjusted GM (95% CI)^b^	*P* value
Only smoke	0.494 (0.386-0.633)	Reference	0.433 (0.324-0.578)	Reference
Only vapor	0.081 (0.048-0.137)	<.001	0.126 (0.077-0.206)	<.001
Neither	0.016 (0.013-0.021)	<.001	0.021 (0.016-0.028)	<.001

Abbreviation: GM, geometric mean.

SI conversion factor: To convert cotinine to nanomoles per liter, multiply by 5.675.

^a^ Serum cotinine concentration used as a biomarker of recent nicotine absorption. Estimates come from log-normal tobit regression models accounting for complex design of the National Health and Nutrition Examination Survey.

^b^ Adjusted model included age, sex, ethnicity, family income, log(body weight), and log(height) as covariates. Adjusted geometric means are marginalized over covariates. Data and analysis code are openly available online.^22^

## Discussion

This cross-sectional study using data drawn from a nationally representative US survey found that, as of 2020, approximately 1 in 5 children was exposed to secondhand tobacco smoke or e-cigarette vapor indoors (mostly secondhand smoke). Children exposed to secondhand e-cigarette vapor only had approximately 84% lower absorption of nicotine than those exposed to secondhand tobacco smoke only. However, children with no secondhand exposure had the lowest nicotine absorption (approximately 97% lower than for secondhand smoke). These large differences remained after adjustment for age, sex, ethnicity, family income, body weight, and height.

Our findings mirror those from a previous study^14^ using silicone wristbands, which absorb nicotine, to measure children’s secondhand exposure from vaping vs smoking. That study found that, compared with children exposed to secondhand tobacco smoke in the home, nicotine levels measured in the wristband were 88% lower in children exposed to e-cigarette vapor only in the home and 98% lower in those exposed to neither. This finding closely aligns with the approximately 84% and 97% lower levels of serum cotinine we identified. Taken together, these studies show that secondhand exposure to nicotine is likely much lower from vaping than smoking, as would be expected from the pharmacokinetic studies showing that e-cigarette users retain the vast majority of nicotine produced while vaping.^7^ Exposure to other harmful toxicants and carcinogens will be lower still, given that these are either absent from e-cigarette aerosol or present in much lower concentrations compared with tobacco smoke.^9,10,11,12^ Nonetheless, vaping inside the home around children should be avoided given that, even though nicotine itself has a limited risk profile, the increased absorption of nicotine from secondhand vapor suggests that the children were also exposed to other potentially harmful excipients from e-cigarettes.

Compared with the amount of nicotine they deliver to users, e-cigarettes also produce much lower levels of toxicants and carcinogens than do cigarettes. Thus, for bystanders, the estimated 80% to 90% lower exposure levels to nicotine from secondhand vapor compared with secondhand smoke is likely to underestimate the reduction in exposure to other harmful substances.^9,10,11,12^

The current results reflect children’s relative secondhand nicotine absorption from smoking vs vaping as these products are used in the general population. Therefore, some of the disparity in absorption could be due to differences in the frequency and intensity with which children are exposed to vaping vs smoking indoors. For instance, if people who vaped indoors did so more often than those who smoked, the estimated 84% lower exposure would be an underestimate of the difference had the children been around equivalent amounts of vaping to smoking. Subsequent studies could examine this by measuring and adjusting for these differences.

### Limitations

The study benefits from using data from a nationally representative of US children, with cotinine used as an objective and extensively validated biomarker of recent secondhand nicotine absorption.^1,19,20,23^ However, there are also several limitations. First, there may be social desirability bias; questions about a child’s secondhand exposure were answered by the proxy respondent (usually the mother or father), who may have been hesitant to report that they allowed their child to be exposed. Thus, some children in the group with no reported exposure may, in fact, have been exposed, which would lead us to overestimate cotinine levels in this group. Second, since the data were collected (2017-2020), there has been a notable shift in the vaping market; modern disposable vaping products have become increasingly popular.^24,25^ Patterns of indoor use and vapor generation may differ for disposable and rechargeable e-cigarettes, so our results will need to be replicated with more recent data. Third, questions about past 7-day secondhand exposure were asked at the initial interview, but blood samples were taken at the mobile examination center visit a few days later. Nonetheless, this issue would affect estimates for secondhand vapor and smoke exposure equally, so is unlikely to bias comparisons between these groups. Fourth, we had insufficient sample size to examine associations stratified by race and ethnicity, household income, or other important equity variables. Furthermore, some of the children who were reported to only have been exposed to secondhand e-cigarette vapor also lived with a tobacco smoker. Excluding these children lowered the geometric mean cotinine level among the secondhand vapor group, but it remained significantly higher than in no exposure group (eTable in Supplement 1), suggesting that some, but not all, of the increased nicotine absorption from secondhand vapor exposure may instead be due to secondhand (or thirdhand) smoke.

## Conclusions

In summary, we found that nicotine absorption is much lower in children who are exposed to secondhand e-cigarette vapor vs secondhand tobacco smoke, but still approximately 5 times higher than in those exposed to neither. This suggests that switching from smoking to vaping indoors may substantially reduce but not eliminate children’s secondhand exposure to nicotine and other noxious substances.

## Notes

Supplement 1. **eTable.** Sensitivity analysis examining nicotine exposure by reported secondhand exposure to tobacco smoke and e-cigarette vapour in children (aged 3–11 years), excluding those in the “only vaping” group who live with a tobacco smoker

Supplement 2.
Data Sharing Statement
