# Supplementary material for: Secondhand Nicotine Absorption From E-Cigarette Vapor vs Tobacco Smoke in Children
Source: JAMA Netw Open. 2024 Jul 11;7(7):e2421246. doi: 10.1001/jamanetworkopen.2024.21246 (PMC11240186; doi:10.1001/jamanetworkopen.2024.21246)
Supplement: Supplement 1. — eTable. Sensitivity analysis examining nicotine exposure by reported secondhand exposure to tobacco smoke and e-cigarette vapour in children (aged 3–11 years), excluding those in the “only vaping” group who live with a tobacco smoker [file jamanetwopen-e2421246-s001.pdf]

Supplemental Online Content

Tattan-Birch H, Brown J, Jackson SE, Jarvis MJ, Shahab L. Secondhand nicotine absorption from e-cigarette vapor vs tobacco smoke in children. *JAMA Netw Open*. 2024;7(7):e2421246. doi:10.1001/jamanetworkopen.2024.21246

eTable. Sensitivity analysis examining nicotine exposure by reported secondhand exposure to tobacco smoke and e-cigarette vapour in children (aged 3–11 years), excluding those in the "only vaping" group who live with a tobacco smoker

This supplemental material has been provided by the authors to give readers additional information about their work.

© 2024 Tattan-Birch et al. *JAMA Network Open.*

eTable. Sensitivity analysis examining nicotine exposure by reported secondhand exposure to tobacco smoke and e-cigarette vapour in children (aged 3–11 years), excluding those in the "only vaping" group who live with a tobacco smoker

	Serum cotinine, ng/ml [95% CI] [1]			
**Secondhand exposure**	**Unadjusted GM**[1]	**P-value**	**Adjusted GM**[1]	**P-value**
**Only smoke**	0.494 [0.386-0.633]	Ref	0.433 [0.324-0.578]	Ref
**Only vapour (not living with smoker)**	0.053 [0.025-0.109]	<.0001	0.082 [0.040-0.168]	<.0001
**Neither**	0.016 [0.013-0.021]	<.0001	0.021 [0.016-0.028]	<.0001

[1] GM = Geometric mean; CI = Confidence interval. Serum cotinine concentration used as a biomarker of recent nicotine exposure. Estimates come from log-normal tobit regression models accounting for complex design of the NHANES survey. Adjusted model included age, sex, ethnicity, family income, log(bodyweight), and log(height) as covariates. Adjusted geometric means are marginalised over covariates. Data and analysis code openly available online (https://osf.io/7z5j6/).

© 2024 Tattan-Birch et al. *JAMA Network Open.*
