# Supplementary material for: Secondhand Nicotine Absorption From E-Cigarette Vapor vs Tobacco Smoke in Children
Source: JAMA Netw Open. 2024 Jul 11;7(7):e2421246. doi: 10.1001/jamanetworkopen.2024.21246 (PMC11240186; doi:10.1001/jamanetworkopen.2024.21246)
Supplement: Supplement 2. — Data Sharing Statement [file jamanetwopen-e2421246-s002.pdf]

Data Sharing Statement

Tattan-Birch. Secondhand Nicotine Absorption From E-Cigarette Vapor vs Tobacco Smoke in Children. *JAMA Netw Open*. Published July 11, 2024.
doi:10.1001/jamanetworkopen.2024.21246

Data
Data available: Yes
Data types: Deidentified participant data
How to access data: Data and analysis code are openly available on the Open Science Framework (https://osf.io/7z5j6/).
When available: With publication

Supporting Documents
Document types: Statistical/analytic code
How to access documents: Data and analysis code are openly available on the Open Science Framework (https://osf.io/7z5j6/).
When available: With publication

Additional Information
Who can access the data: Anyone requesting the data.
Types of analyses: Any purpose.
Mechanisms of data availability: Open download from an online repository.
